# Sirtuin-3 promotes osteoclast maturation and bone loss by regulating mitochondrial ROS production during ionizing radiation exposure

**DOI:** 10.1093/jbmrpl/ziaf092

**Published:** 2025-05-19

**Authors:** Gareeballah Osman Adam, Kimberly K Richardson, Ankita Chalke, Qiang Fu, Jeff D Thostenson, Hutomo Tanoto, Yuxiao Zhou, Nukhet Aykin-Burns, Ha-Neui Kim

**Affiliations:** Center for Musculoskeletal Disease Research, University of Arkansas for Medical Sciences, Little Rock, AR 72205, United States; Division of Endocrinology, Department of Internal Medicine, University of Arkansas for Medical Sciences, Little Rock, AR 72205, United States; Center for Musculoskeletal Disease Research, University of Arkansas for Medical Sciences, Little Rock, AR 72205, United States; Division of Endocrinology, Department of Internal Medicine, University of Arkansas for Medical Sciences, Little Rock, AR 72205, United States; Center for Musculoskeletal Disease Research, University of Arkansas for Medical Sciences, Little Rock, AR 72205, United States; Division of Endocrinology, Department of Internal Medicine, University of Arkansas for Medical Sciences, Little Rock, AR 72205, United States; Center for Musculoskeletal Disease Research, University of Arkansas for Medical Sciences, Little Rock, AR 72205, United States; Division of Endocrinology, Department of Internal Medicine, University of Arkansas for Medical Sciences, Little Rock, AR 72205, United States; Center for Musculoskeletal Disease Research, University of Arkansas for Medical Sciences, Little Rock, AR 72205, United States; Department of Biostatistics, University of Arkansas for Medical Sciences, Little Rock, AR 72205, United States; Department of Mechanical Engineering, Texas A&M University, College Station, TX 77840, United States; Department of Mechanical Engineering, Texas A&M University, College Station, TX 77840, United States; Division of Radiation Health, Department of Pharmaceutical Sciences, University of Arkansas for Medical Sciences, Little Rock, AR 72205, United States; Center for Musculoskeletal Disease Research, University of Arkansas for Medical Sciences, Little Rock, AR 72205, United States; Division of Endocrinology, Department of Internal Medicine, University of Arkansas for Medical Sciences, Little Rock, AR 72205, United States

**Keywords:** SIRT3, osteoclast, bone resorption, mitochondria, ionizing radiation, ROS

## Abstract

Ionizing radiation (IR) exposure leads to mitochondrial alterations in osteoclasts and osteoblasts, contributing to musculoskeletal disintegration. Despite this, the mechanisms controlling mitochondrial activity in bone cells during IR exposure-associated bone disorders remain underexplored. Sirtuin-3 (SIRT3), a NAD-dependent mitochondrial deacetylase, is essential for the enhanced mitochondrial function in osteoclasts and the increased bone resorption observed in osteoporosis. However, it is still unclear whether and how SIRT3 drives IR exposure-induced bone disorders. Here, we show that deletion of *Sirt3* greatly attenuated the IR exposure-induced loss of bone mass in young adult mice. This effect was associated with impaired osteoclast maturation and function, thus suppressing excessive bone resorption. IR exposure also increased mitochondrial activity and ROS production in osteoclasts. Deletion of *Sirt3* abrogated these effects of IR exposure. The levels of mitochondrial superoxide dismutase 2 (SOD2), a major component of the metabolic machinery that handles ROS in the mitochondrial matrix, were significantly increased in osteoclasts by RANKL with an identical pattern as SIRT3. Deacetylation of lysine 68 of SOD2 enhanced the formation of giant osteoclasts and increased mitochondrial ROS production, mimicking the effects of IR exposure. Inhibition of mitochondrial ROS production via Mito-TEMPO recapitulated the effects of *Sirt3* deletion on osteoclast maturation and mitochondrial activity during IR exposure. These findings demonstrate that SIRT3 plays an essential role in IR exposure-induced bone resorption by promoting deacetylation in osteoclast mitochondria. Understanding the mechanisms of mitochondrial quality control and protein acetylation in osteoclasts could pave the way for developing novel strategies to counteract IR exposure-associated bone disorders.

## Graphical Abstract

**Graphical Abstract:**
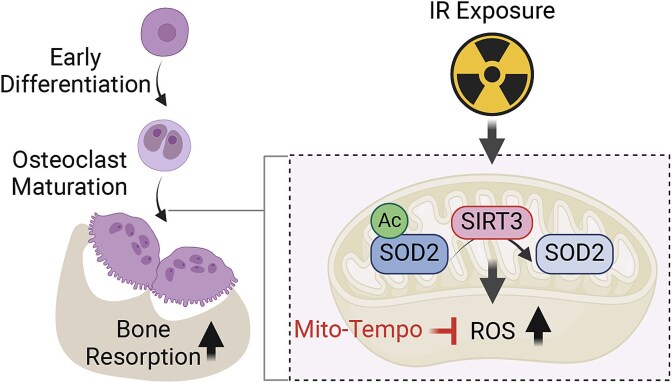

## Introduction

Osteoclasts are large, motile, multinucleated, bone-resorbing cells derived from precursor cells of the macrophage lineage that reside in the hematopoietic bone marrow. Whereas macrophage CSF (M-CSF) binds to its receptor, c-Fms, on the surface of osteoclast progenitor cells and acts as a survival factor precursor,^1^^,^^2^ the initial step of osteoclast differentiation is driven by the binding of the RANKL to its receptor, RANK, on the surface of osteoclast progenitor cells to generate pre-osteoclasts that are potentially capable of resorbing bone, but still immature.^3^^,^^4^ These pre-osteoclasts then fuse with each other to form mature osteoclasts that adhere tightly to the bone surface targeted for “eating.” During adulthood, this primary function of mature osteoclasts coupled to bone formation rejuvenates the skeleton and is essential for bone remodeling.^5^^,^^6^ With pathological or physiological changes, such as aging, inflammation, or ionizing radiation (IR) exposure, the amount of bone resorbed by osteoclasts is not fully replenished with new bone formed by osteoblasts, and this excessive bone resorption is the primary cause of low bone mass and increased fracture risk.^7^^,^^8^ Although much is known about the signals and molecules regulating early osteoclast differentiation, we still do not understand the molecular mechanisms by which pre-osteoclasts fuse with each other and by which the large, multinucleated osteoclasts move and digest bone matrix. We expect that understanding how osteoclasts satisfy their high energy demands may clarify how osteoclasts perform their multinucleation and bone resorptive activity.

The prevailing idea for the pathogenesis of bone disorders has long been that IR exposure is the seminal mechanism leading to the development of severe bone loss; however, the mechanism leading to acute activation of osteoclasts after IR exposure remains unknown. IR exposure constitutes an occupational risk (eg, astronauts and military personnel). Those at risk are exposed to low doses and continual IR. Unfortunately, current antiresorptive or anabolic therapies (eg, bisphosphonates and parathyroid hormone) focus on targeting cells of the bone remodeling unit to prevent osteopenia and have not significantly decreased the risk for fractures in patients who received radiotherapy.^9^^,^^10^ For these reasons, identifying novel mechanisms that impair IR exposure-induced bone damage may aid in preventing insufficiency fractures (commonly associated with osteoporosis).

Mitochondria act as the power plants of the cell and are constantly maintained via quality control processes that include fusion and fission of the tubular mitochondrial network, mitochondrial biogenesis, and the elimination of unwanted mitochondria by mitophagy (ie, mitochondria-specific autophagy).^11^ It is essential to maintain healthy mitochondria to achieve energy production, fatty acid oxidation, and other functions that are ultimately served by oxidative phosphorylation and electron transport in the inner mitochondrial membrane. Oxidative phosphorylation produces mitochondrial ROS as a by-product; ROS can be important signaling molecules, but when produced in excess, ROS damage cells and contribute to disease and aging.^12^^,^^13^ Although it is well established that RANKL stimulates the production of mitochondrial ROS, which contribute to osteoclastogenesis,^14^ we still do not understand the molecular mechanisms that control mitochondrial ROS and metabolic function in osteoclasts or how changes to these mechanisms result in skeletal disorders.

We and others showed that the primary mitochondrial protein deacetylase sirtuin-3 (SIRT3) promotes the excessive bone resorption that results from estrogen deficiency and aging.^15–18^ Osteoclast formation and bone resorption are promoted by the deacetylation of specific mitochondrial quality control proteins, maintaining the proper function of osteoclast mitochondria.^15^^,^^18^^,^^19^ More recently, we showed that *Sirt3* mRNA levels and enzymatic activity increased significantly in cultured osteoclasts from irradiated mice, resulting in increased mitochondrial respiration and ROS generation.^20^^,^^21^ We proposed that IR exposure induces trabecular, but not cortical, bone loss in young adult mice by promoting osteoclast differentiation and activity, with SIRT3 potentially responsible for this effect. However, our previous work utilized cultured osteoclast progenitors. Thus, it remains necessary to determine whether SIRT3 influences the differentiation and activation of osteoclasts in vivo during IR exposure, as observed in aged or ovariectomized (estrogen-deficient) mice.^15–18^ Furthermore, the mitochondrial proteins and associated post-translational modifications, especially lysine deacetylation, that are targeted by SIRT3 have not been identified in osteoclasts during IR exposure-induced bone loss. We show herein that deletion of *Sirt3* in mice or pharmacologic inhibition of mitochondrial quality control in osteoclasts prevented the IR exposure-induced loss of bone mass and associated increase in osteoclast maturation and function. We also propose that superoxide dismutase 2 (SOD2), the mitochondrial manganese superoxide dismutase (MnSOD), may be a target of SIRT3 in osteoclasts.

## Materials and methods

### Animals

Animals were obtained from The Jackson Laboratory and housed at the University of Arkansas for Medical Sciences (UAMS; 4 to 5 per cage) with standard chow and water ad libitum. Mice heterozygous for the *Sirt3* mutant allele (B6/Sv129 mixed background) were intercrossed to produce male homozygous null mice (*Sirt3*-KO), as described before.^15^ Cohorts of *Sirt3*-KO and their littermate controls (WT) were aged up to 6 mo. Mice were exposed to weekly 0.33 Gy total-body irradiation (TBI) for either 12 or 6 fractions with 1 fraction per wk using a J.L. Shepherd Mark 1 Model 68A ^137^Cs γ irradiator at the same time each day. Sham controls (WT or *Sirt3*-KO) underwent the same protocol without irradiation.

### DXA and micro-computed tomography

BMD of lumbar spine, the left femur, or the whole body (except the head) was measured with DXA using a PIXImus densitometer (GE Lunar); mice were anesthetized with 2% isoflurane. Bone architecture was measured with a MicroCT40 (Scanco Medical); the left femora and vertebrae (L5) were dissected, cleaned, fixed in Millonig’s phosphate buffer (Leica Biosystems), and gradually dehydrated in 100% ethanol. Bone specimens were subjected to micro-computed tomography (micro-CT) imaging utilizing the Scanco Medical MicroCT40 instrument under defined scanning parameters. A voxel size of 12 μm was used, consistent with guidelines for micro-CT imaging in bone morphometry analysis. The resulting voxel data (1024 × 1024 pixel matrices per planar stack) were integrated into 3D representations and analyzed with the manufacturer’s software (Scanco Eval Program v6.0). Prior to the determination of cortical and trabecular parameters, a Gaussian filter (sigma = 0.8, support = 1) was applied to all the imaged scans. Recommended grayscale thresholds for bone tissue set forth by the American Society for Bone and Mineral Research^22^ were implemented, with a value of 200 mg cm^−3^ for cortical bone and 220 mg cm^−3^ for trabecular regions of interest. Cortical morphology was quantified at the femoral midshaft via assessment of 18-23 slices and at the distal metaphysis using 50 slices spanning coordinates 300-350. Total area, medullary area, and circumferential measurements were calculated from these defined cortical sites. In the vertebral bones, contours were drawn from the rostral to the caudal growth plate to obtain 200 slices (12 μm/slice), and bone outside the vertebral body plate was excluded. For trabecular analysis at the distal femur, 151 slices were evaluated, starting 8-10 slices from the growth plate and extending proximally; this omitted the primary spongiosa-containing region so as to avoid confounding metrics. A contouring technique was used for demarcating trabecular regions within each slice, explicitly excluding cortical bone. Subsequent trabecular morphometric quantification relied on a distance-transform method without a priori rod or plate assumptions for the bone geometry.

### Mechanical testing

One lumbar spine (L1) specimen was selected from each group, ensuring its stiffness was closest to the mean stiffness within the group. Compression testing was conducted on the selected specimens using a loading stage (CT5000, Deben) coupled with a micro-CT scanner. A compressive load of 42 g was applied to the top of each L1 vertebral body through a thin layer of polymethyl methacrylate (PMMA). Micro-CT scans were performed both before loading and after the bone had stabilized under compressive loading. CT images were acquired at a voltage of 90 kV and a current of 133 μA, with an isometric voxel size of 10. 67 μm (Xradia 620 Versa, Zeiss). The 3D full-field displacement within each L1 vertebral body was calculated by performing digital volume correlation (DVC) analysis on the micro-CT images captured under no-load and loaded conditions, using DaVis software (LaVision). Multiple passes of correlation were carried out with progressively smaller isometric correlation window sizes. The final pass used a window size of 682.9 μm, with a 50% overlap between adjacent correlation windows. Displacement vectors with correlation values below 0.5 or peak ratios below 2.5 were excluded and replaced by interpolation of neighboring vectors. To eliminate the effects of rigid body movement, the images were rigidly translated to align the distal end of the L1 vertebral body in both the loaded and no-load images. The strain values inside the L1 vertebral body were calculated by numerically differentiating the displacement field and were not influenced by the PMMA topping. The measured strain fields were mapped onto the bone microstructures using volume rendering in Avizo software. The minimum principal strains within each correlation window were extracted and exported for statistical analysis.

### Bone histology

L_2_-L_4_ vertebrae were dissected and placed in 10% Millonig’s Neutral Buffered Formalin with 5% sucrose fixative, dehydrated with ethanol, and kept in 100% ethanol until analysis. Vertebrae were embedded in methyl methacrylate (Sigma-Aldrich). Bone histomorphometry was performed using standard terminology defined by the Histomorphometry Nomenclature Committee of the American Society for Bone and Mineral Research.^23^ Longitudinal sections (5 μm thick) were cut in the medial–lateral plane using a rotary microtome. Osteoclasts were identified by staining for tartrate-resistant acid phosphatase (TRAP) activity using naphthol AS-MX phosphate and Fast Red TR salt (Sigma-Aldrich). Adjacent sections were stained with 0.3% toluidine blue (pH 3.7) to visualize osteoblasts, osteoid, and cement lines. Histomorphometric measurements were obtained using an Olympus BX53 microscope equipped with an Olympus DP73 digital camera and Osteomeasure software v4.1.0.2 (OsteoMetrics). One section per sample was analyzed in a blinded fashion.

### CTx and P1NP ELISA

Whole blood was collected from mice via retro-orbital bleeding into Eppendorf tubes. Samples were kept on ice for 30 min prior to centrifugation at 10 000 × *g* for 10 min at 4 °C to collect serum. Serum concentrations of CTx and P1NP were quantified by ELISA using commercially available kits (RatLaps CTX-I ELISA kit or Rat/Mouse PINP EIA kit; Immunodiagnostic Systems) according to the manufacturer’s protocol. Absorbance was measured on a microplate reader, and sample concentrations were determined by interpolation from a standard curve.

### Osteoclast differentiation

Bone marrow-derived macrophages (BMMs) were isolated as previously described.^14^ Briefly, whole bone marrow cells were cultured overnight in α-MEM supplemented with 10% FBS and 1% penicillin-streptomycin in the presence of 10 ng mL^−1^ M-CSF (R&D Systems). Nonadherent cells were collected and further cultured in M-CSF (30 ng mL^−1^) for 3 to 4 d. Adherent BMMs were harvested as osteoclast precursors. To generate mature osteoclasts, BMMs were cultured for 4-5 d in medium containing M-CSF and the RANKL (30 ng mL^−1^; R&D Systems). Multinucleated osteoclasts were detected with TRAP staining using a commercial kit (Sigma-Aldrich) per the manufacturer’s protocol and counted by light microscopy.

### Bone resorption activity assay

BMMs were cultured for 4-5 d in a medium supplemented with M-CSF and RANKL (30 ng mL^−1^) to induce osteoclast differentiation. The bone resorption activity assay was performed using a calcium phosphate-coated 48-well plate (Cosmo Bio). BMMs were seeded at a density of 5 × 10^4^ cells per well. Bone resorption activity was assessed by measuring the fluorescence intensity of the culture medium. The excitation and emission wavelengths used for fluorescence detection were identical to those of FITC. After the resorption assay, the remaining medium was carefully removed from each well, and the wells were treated with 5% sodium hypochlorite for 3-5 min. The plates were then washed with water and air dried. To visualize bone resorption area, images of resorption pits in each well were captured using a microscope.

### Mitochondrial respiration and cellular bioenergetics

Oxygen consumption rate (OCR) analyses were conducted utilizing a Seahorse XF96 analyzer (Agilent). Osteoclasts were previously cultured in Seahorse XF 96 culture plates with RANKL for 3 d prior to assay. On the day of metabolic analysis, cells were changed into Seahorse XF medium and maintained at 37 °C in a non-CO_2_ incubator per the standardized protocol.^24^ Baseline cellular respiration was initially recorded, followed by serial injection of modulators of mitochondrial respiration through instrument ports. To determine ATP-linked OCR, oligomycin (Sigma-Aldrich) was injected at 10 μg mL^−1^ final concentration to block ATP synthase activity. Maximal respiratory capacity was elicited using the uncoupling agent carbonyl cyanide-4-(trifluoromethoxy) phenylhydrazone (Sigma-Aldrich) at 10 μm. Finally, to quantify nonmitochondrial oxygen consumption, the electron transport chain inhibitors antimycin A and rotenone (Sigma-Aldrich) were introduced at 10 μm. Using these data, additional mitochondrial parameters were calculated, including basal respiration, spare respiratory capacity, proton leak, and coupling efficiency.

### Western blot

BMMs were cultured with M-CSF and RANKL. On day 2, the cells were irradiated at a dose of 0.33 Gy. On day 3, the cultured cells were washed twice with PBS and lysed with buffer containing 20 mm Tris–HCL, 150 mm NaCl, 1% Triton X-100, a protease inhibitor mixture, and a phosphatase inhibitor cocktail (Sigma-Aldrich) on ice for 30 min. Then, the cells were centrifuged at 11 200 × *g* for 15 min at 4 °C, and the supernatant was collected in a new tube. The protein concentration was measured using a DC protein assay kit (Bio-Rad); 30-40 μg of protein was extracted per sample. The protein samples were separated on SDS-PAGE gels and transferred electrophoretically to polyvinylidene difluoride membranes (Millipore Sigma). The membranes were blocked with 5% fat-free milk/Tris-buffered saline for 120 min and incubated with a primary antibody, followed by a secondary antibody conjugated to horseradish peroxidase. Immunoblot analyses used mouse monoclonal primary antibodies directed against the following target proteins: NFATc1 (Santa Cruz Biotechnology, sc-7294; 1:500 dilution), mitofusin 2 (Abcam, ab56889; 1:1000), and BCL2/adenovirus E1B 19 kDa interacting protein 3 (BNIP3) (Abcam, ab10433; 1:1000). Additionally, rabbit monoclonal antibodies were used to detect SIRT3 (Cell Signaling Technology, 5490; 1:1000), succinate dehydrogenase complex flavoprotein subunit A (SDHA) (Cell Signaling Technology, 11998; 1:1000), acetylated superoxide dismutase 2 (Ac-SOD2) at lysine 68 (Abcam, ab137037; 1:1000), and NIP3-like protein X (NIX) (Cell Signaling Technology, 12396; 1:1000). Rabbit polyclonal antibodies were used to detect the total protein of SOD2 (Abcam, ab13533; 1:1000). β-Actin was used as a loading control (Santa Cruz Biotechnology, sc-81178; 1:2000). Antibody–antigen complexes were visualized with enhanced chemiluminescence reagents (MilliporeSigma) and quantified with imaging under standardized conditions (VersaDoc, Bio-Rad).

### Mitochondrial ROS production

Mitochondrial ROS were detected with the MitoSOX Red reagent (M36008, Thermo Fisher Scientific) according to the manufacturer’s protocol. Briefly, BMMs were seeded into 96-well black microplates and treated with RANKL for 3 d. The medium was replaced with the assay medium containing 3 μm MitoSOX reagent and incubated for 30 min at 37 °C. Fluorescence was measured kinetically at 510/580 nm excitation/emission wavelengths using a Cytation 5 microplate reader (BioTek Instruments) over 120 min to assess the rate of MitoSOX oxidation.

### Functional analysis of the effects of site-directed SOD2 acetylation mutations

To investigate the role of SOD2 deacetylation in *Sirt3*-mediated osteoclast maturation and mitochondrial metabolism in vitro, we generated WT and deacetylation-mimicking SOD2 cDNA constructs. These constructs were developed in collaboration with Biocytogen by subcloning the respective SOD2 mutants into a modified version of the pUC19 vector, which included a Flag tag. We engineered constitutively deacetylated mutants of the SOD2 protein by mutating lysine residues at acetylation sites K68 and K122 to arginine, thereby preventing acetylation and mimicking a deacetylated state. The constructs were verified by DNA sequencing. Ralph and Athymic White (RAW) 264.7 murine macrophage cells were seeded in 6-well plates 24 h prior to transfection. The cells were then transfected with the SOD2 mutants using Lipofectamine Plus reagent (Invitrogen) following the manufacturer’s instructions. After 20 min of incubation at room temperature in serum-free medium, the cells were washed and cultured in α-MEM complete media for 24 h. RANKL (30 ng mL^−1^) was added to the cultures, and the cells were incubated for an additional 5 d. To further investigate the functional role of SOD2 mutants, we subcloned the same SOD2 mutant constructs into the pMX-IRES-puro retroviral vector. Retrovirus packaging was carried out by transient transfection of these vectors into Plat-E retroviral packaging cells. For retroviral infection, BMMs were incubated with polybrene (6 μg mL^−1^) and culture supernatants were collected from Plat-E cells transfected with the retroviral vectors. Infected BMMs were further cultured in M-CSF (30 ng mL^−1^) for 24 h, followed by experimental treatments as indicated. Multinucleated osteoclasts were detected using TRAP staining (Sigma-Aldrich) according to the manufacturer’s protocol and counted under a light microscope. Overexpression of each construct was confirmed by Western blot analysis, utilizing Flag tag detection (data not shown).

### Statistics

For serum CTx and P1NP data, repeated-measures ANOVA models were considered with main effects of genotype and month, interactions of the main effects, and month as the categorical or continuous variable, with a possible quadratic term. After examining the significance of effects and model-fit statistics, models with categorical effects of genotype, month, and their interaction were chosen for both models. These analyses were conducted with SAS v9.4. All the other analyses were conducted with GraphPad Prism 10.0 (GraphPad Software). Data were assessed for normality and equal variance prior to analysis. Differences between experimental groups were determined with 1- or 2-way ANOVA, with statistical significance defined as *p* < .05. For comparisons between only 2 experimental groups, an unpaired two-tailed Student’s *t*-test was used. A *p* < .05 was considered statistically significant.

### Study approval

The Institutional Animal Care and Use Committees of the University of Arkansas for Medical Sciences reviewed and approved all the studies involving mice.

## Results

### Ionizing radiation exposure-associated loss of trabecular bone mass is prevented in *Sirt3*-null mice

**Figure 1:**
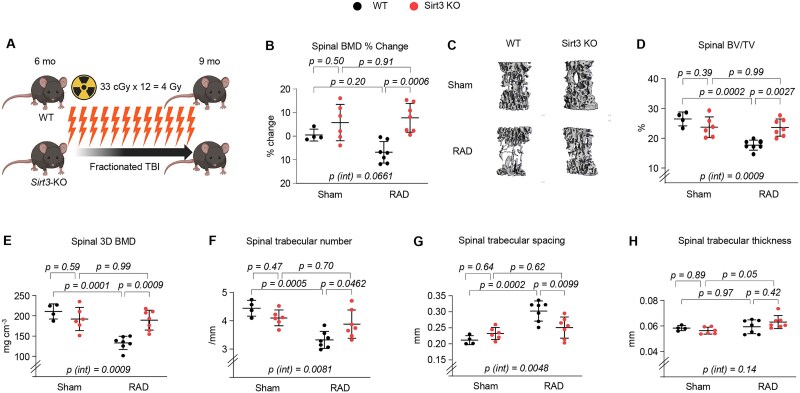
Deletion of *Sirt3* prevents IR exposure–induced trabecular bone loss in the spine. (A) Schematic representation of the scheduling of total body irradiation (TBI) exposures. (B) Percentage change in BMD by DXA 1 d before IR exposure and before sacrifice. (C-H) Imaging and quantification of spinal bones from sham and irradiated WT and *Sirt3*-KO male mice with micro-CT after euthanasia (*n* = 4-7 animals/group). (C) Representative 3D trabecular bone images of the spine. (D) Bone volume over tissue volume (BV/TV), (E) 3D BMD after last irradiation, (F) trabecular number, (G) spacing, and (H) thickness of trabecular bone in the spine measured with micro-CT in irradiated mice and sham controls. Line and error bars represent mean ± SD. Statistical significance was determined using 2-way ANOVA.

**Figure 2:**
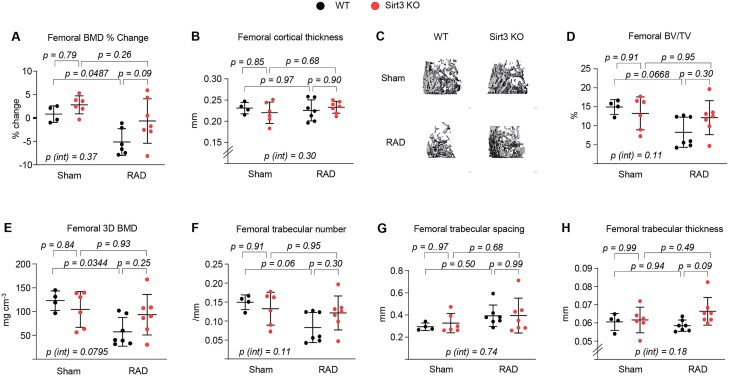
Deletion of *Sirt3* attenuates IR exposure–induced trabecular bone loss in the femur. (A-H) Imaging and quantification of cortical and trabecular bones from sham and IR-exposed WT and *Sirt3*-knockout male mice with micro-CT after euthanasia (*n* = 4-7 animals/group). (A) Percentage change in BMD by DXA 1 d before IR exposure and before sacrifice. (B) Midshaft cortical bone thickness. (C) Representative 3D trabecular bone images of femur. (D) Bone volume over tissue volume (BV/TV), (E) 3D BMD after last irradiation, (F) trabecular number, (G) spacing, and (H) thickness of trabecular bone in the femur measured with micro-CT in irradiated mice and sham controls. Line and error bars represent mean ± SD. Statistical significance was determined using 2-way ANOVA.

To determine the long-term effects of low-dose IR exposure on bone health, WT and *Sirt3*-KO mice were exposed to 12 fractions of 0.33 Gy TBI per week for 12 wk (Figure 1A). Ionizing radiation exposure caused a decrease of BMD in the spine and femur of WT mice, as determined by DXA (Figures 1B and 2A). However, deletion of *Sirt3* strongly attenuated the loss of spine and femur BMD with IR exposure, although this effect was more pronounced in the spine. As expected,^20^ analysis of the bone microarchitecture with micro-CT confirmed that WT mice had lower trabecular bone volume and 3D BMD at both the spine and femur (Figures 1C-E and 2C-E), but there were no changes in cortical thickness (Figure 2B). The decrease in trabecular bone mass in the irradiated WT mice was due to a decrease in trabecular number and an increase in trabecular spacing, but no changes were observed in trabecular thickness (Figures 1F-H and 2F-H). The IR exposure–induced loss of trabecular bone (trabecular bone volume) was attenuated in *Sirt3*-KO mice (Figures 1C-E and 2C-E), but this effect was more pronounced in the spine than in the femur (spine: *p* = .0009 for interaction; femur: *p* = .11 for interaction). This preservation was associated with the return of the trabecular number to the WT value and suppressed trabecular spacing (Figure 1F-G). Interestingly, while the magnitude of compressive strain in spinal bones was not significantly altered in irradiated mice compared to sham controls, our DVC analysis revealed a trend toward stronger bones in *Sirt3*-KO mice under IR exposure. This enhanced load-bearing function in *Sirt3*-KO mice, characterized by a reduced magnitude of compressive strain under identical mechanical loading, suggests that SIRT3 contributes to bone fragility in this context (Figure S1). Taken together, these results indicate that SIRT3 contributes to low-dose IR exposure–related bone loss in young adult mice and that its contribution is greater in the spine than in the femur. Our findings also suggest that distinct mechanisms are responsible for the skeletal damage in the trabecular versus cortical bone compartment during IR exposure.

### Deletion of *Sirt3* suppresses bone resorption activity without altering osteoclast number in vivo

**Figure 3:**
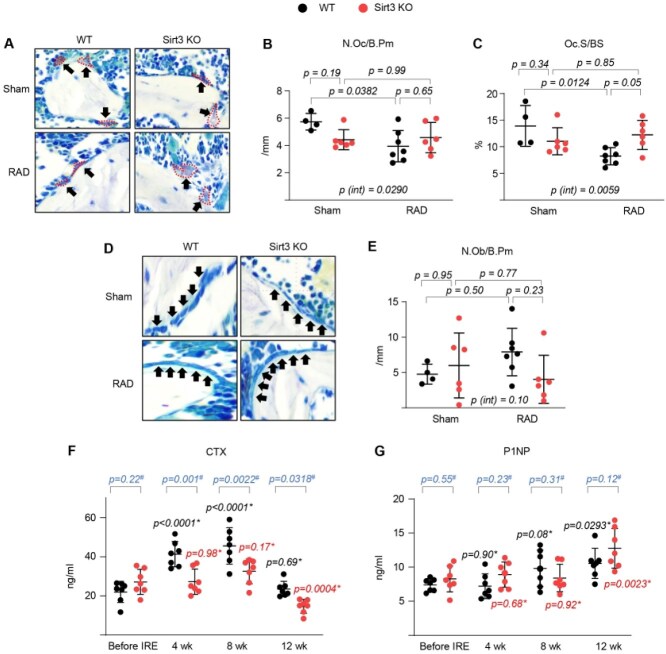
Deletion of *Sirt3* reduces IR exposure–induced bone resorption activity without affecting osteoclast number. Representative photomicrographs of (A) osteoclasts and (B) number and (C) surface of osteoclasts per trabecular bone surface of nondecalcified spine sections stained for TRAP activity from IR-exposed WT and *Sirt3*-knockout male mice (*n* = 4-7 animals/group). representative photomicrographs of (D) osteoblasts and (E) number of osteoblasts per trabecular bone surface of nondecalcified spine sections (*n* = 4-7 animals/group). (F and G) ELISA of collagen degradation product (CTx) and P1NP in serum of WT and *Sirt3*-knockout male mice before and after irradiation (*n* = 4-7 animals/group). (B-E) statistical significance was determined using 2-way ANOVA. (F and G) Repeated-measures ANOVA was used to determine *p* values : ^*^ between irradiated mice and nonirradiated controls in the same genotype or # between WT and *Sirt3*-KO mice at the same time point. Line and error bars represent mean ± SD.

Ionizing radiation exposure–related bone loss is associated with increased osteoclast number and increased bone resorption and/or decreased osteoblast number and decreased bone formation.^25–28^ Specifically, mice exposed to low doses of radiation exhibit trabecular bone loss, but not cortical bone loss. The trabecular bone loss is associated with an increase in bone resorption, but not an increase in osteoclast number.^20^^,^^21^^,^^29^ To further explore the cellular mechanisms underlying the protective effect of *Sirt3* deletion on IR exposure-induced bone loss, we first performed histologic analysis to evaluate osteoclast and osteoblast numbers at the trabecular bone surface of the spine. As expected,^20^ IR exposure had no effect on the osteoblast number in WT mice, and the osteoclast number or surface was not increased or even slightly decreased by IR exposure (Figure 3A-E). Furthermore, histomorphometric quantification of osteoclasts and osteoblasts revealed that irradiated *Sirt3*-KO mice showed no differences in osteoclast or osteoblast counts compared to irradiated WT mice. Unexpectedly, the deletion of *Sirt3* tended to increase the osteoclast surface area in irradiated mice (Figure 3C). Serum levels of CTx, a bone resorption marker, peaked in irradiated control mice at approximately week 8 of IR exposure, consistent with low bone mass. However, *Sirt3*-KO mice had much lower serum levels of CTx compared to WT mice, with bone resorption not elevated at all in *Sirt3*-KO mice in response to IR exposure (Figure 3F). Ionizing radiation exposure caused a steady increase in serum levels of P1NP, a bone formation marker in irradiated WT mice, and the deletion of *Sirt3* did not affect it (Figure 3G).

Because peak bone resorption occurred around week 8 of IR exposure, or possibly before the completion of 8 fractions of TBI (Figure 3F), it remains possible that the number of osteoclasts could increase during this period. Therefore, we investigated whether early IR exposure in young adult mice is sufficient to induce bone loss accompanied by a change in osteoclast number and whether deletion of *Sirt3* prevents this. Mice were exposed to 6 fractions of 0.33 Gy TBI per week for 6 wk (Figure S2A). As expected, 6 fractions of TBI activated bone resorption in WT mice, but this was attenuated by the deletion of *Sirt3*, as indicated by the relative levels of serum CTx (Figure S2B). Importantly, early IR exposure in young adult mice was not sufficient to induce trabecular bone loss (Figure S2C) or increase the osteoclast number in the spine (Figure S2D). However, the deletion of *Sirt3* tended to increase the osteoclast number per osteoclast perimeter in irradiated mice (Figure S2E), indicating a smaller osteoclast size. This finding suggests that trabecular bone loss in young adult mice is primarily driven by an increase in osteoclast maturation and function, rather than an increase in osteoclast number, during late-stage IR exposure-related bone loss.

### Deletion of *Sirt3* causes mitochondrial dysfunction in osteoclasts after IR exposure

**Figure 4:**
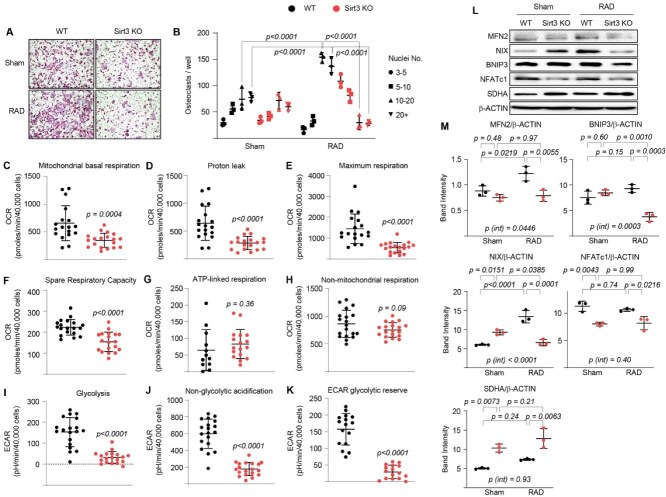
Deletion of *Sirt3* decreases osteoclast maturation and respiration following IR exposure. Bone marrow-derived macrophages (BMMs) were harvested from 6-mo-old male *Sirt3*-KO and WT littermate mice, cultured with M-CSF and RANKL for 2 d and then exposed to a single 0.33 Gy dose of irradiation for an additional (A and B) 2 d or (C-K) 24 h. (A) Representative images and (B) number of TRAP-positive multinucleated osteoclasts derived from irradiated and sham-operated BMMs from WT and *Sirt3*-KO mice. All the cultures were completed in triplicate. Statistical significance was determined with 2-way ANOVA. (C-K) Different parameters of mitochondrial and nonmitochondrial respiration per cell in irradiated cells derived from WT and *Sirt3*-KO mice (*n* = 12-19 wells/group). Line and error bars represent mean ± SD. Statistical significance was determined using a *t-*test. (L) Protein levels were assayed with Western blot and (M) expression levels as the indicated ratio (triplicate cultures). Line and error bars represent mean ± SD. Two-way ANOVA was used to determine *p* values. All the measurements were performed in cultured BMMs pooled from 4 to 5 mice per group and repeated at least twice.

To characterize the molecular mechanism by which bone resorption decreases in *Sirt3*-KO mice, we investigated whether pre-osteoclasts lacking *Sirt3* exhibit abnormal behavior during osteoclastogenesis. Bone marrow macrophages from nonirradiated WT and *Sirt3*-KO mice were cultured in the presence of M-CSF and RANKL. Then, pre-osteoclasts were subjected to a single dose (0.33 Gy) of irradiation to examine the effects of low-dose IR exposure on osteoclast maturation. As expected,^20^ we observed increased osteoclast size and spreading following IR exposure in WT mice. However, macrophages from *Sirt3*-KO mice failed to form multinucleated giant osteoclasts (with more than 10 nuclei) and instead formed a higher number of mononucleated osteoclasts and smaller multinucleated osteoclasts (with fewer than 5 nuclei) compared to those from WT mice (Figure 4A and B). Consistent with this, we have previously found that *Sirt3*-KO osteoclasts resorb less of the biomimetic surface than WT osteoclasts in the presence of IR exposure,^20^ indicating that SIRT3 promotes osteoclast maturation and function during IR exposure–related bone loss. These results were similar to those obtained using cells from aged *Sirt3*-KO mice.^15^

Ionizing radiation exposure increases SIRT3 activity and mitochondrial respiration in osteoclasts.^20^^,^^21^ Here, we examined whether SIRT3 alters mitochondrial activity in osteoclasts by performing extracellular flux analysis. Mitochondrial basal respiration was strongly diminished in *Sirt3*-KO osteoclasts after IR exposure. Deletion of *Sirt3* also decreased proton leak, maximum respiration (ie, maximal electron transport chain activity), and spare respiratory capacity (ie, the difference between maximum OCR and basal OCR) (Figure 4C-F). However, *Sirt3*-KO osteoclasts exhibited no difference in ATP-linked respiration and nonmitochondrial respiration in the presence of IR exposure compared to WT osteoclasts (Figure 4G and H). *Sirt3*-KO cells also exhibited a lower extracellular acidification rate (ECAR) than WT cells (Figure 4I-K). The decrease in ECAR likely reflected an increase in pyruvate entering the tricarboxylic acid (TCA) cycle, resulting in suppression of lactate production.

We next investigated whether the deletion of *Sirt3* altered the expression of proteins involved in mitochondrial quality control, including mitophagy. Consistent with increased osteoclast maturation, IR exposure elevated the expression of mitochondrial quality control proteins, including MFN2, an essential protein responsible for tethering adjacent mitochondria or mitochondria to the endoplasmic reticulum and promoting osteoclast maturation.^30^ However, in *Sirt3*-KO osteoclasts, protein levels of MFN2 were reduced following IR exposure, as were the levels of NIX and BNIP3 (Figure 4L and M), both markers of SIRT3-mediated mitophagy.^15^^,^^31^ Ionizing radiation exposure did not alter the protein levels of NFATc1—a key upstream activator of osteoclastogenesis^4^; however, its expression was suppressed in *Sirt3*-KO cells independently of radiation. Interestingly, the protein levels of factors associated with the electron transport chain, such as SDHA (complex II), were not reduced but instead showed a slight increase following *Sirt3* deletion under IR exposure. Taken together, these findings suggest that SIRT3 is essential for maintaining healthy mitochondria in osteoclasts during IR exposure–related bone loss.

### Deletion of *Sirt3* decreases mitochondrial ROS production in osteoclasts after IR exposure

**Figure 5:**
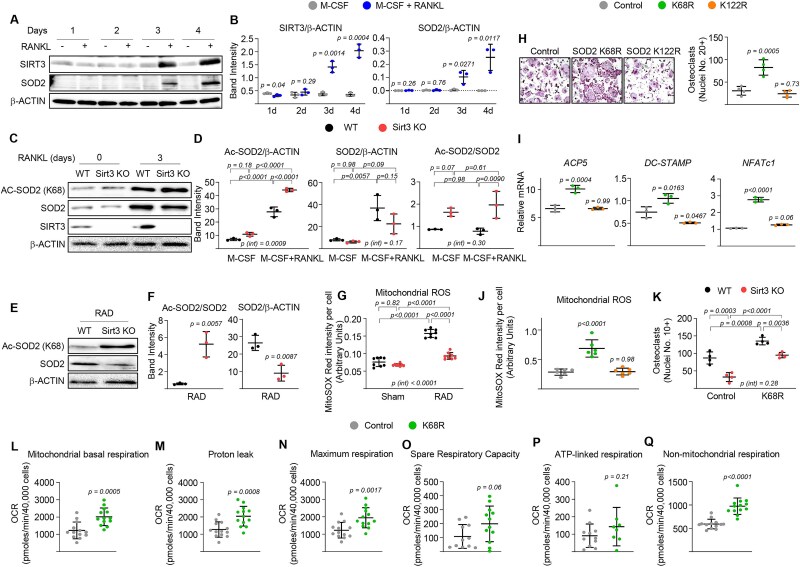
Deacetylation of SOD2 at lysine 68 by SIRT3 promotes osteoclastogenesis. (A and B) Bone marrow-derived macrophages (BMMs) were harvested from 6-mo-old male C57BL/6 mice and cultured with M-CSF and RANKL for the indicated days. (A) Protein levels were assayed with Western blot and (B) expression levels as the indicated ratio (triplicate cultures). (C and D) BMMs were harvested from 6-mo-old male *Sirt3*-KO and WT littermate mice and cultured with M-CSF and RANKL for the indicated days. (C) Protein levels were assayed with Western blot and (D) expression levels as the indicated ratio (triplicate cultures). (E-G) BMMs were harvested from 6-mo-old male *Sirt3*-KO and WT littermate mice, cultured with M-CSF and RANKL for 2 d, and then exposed to a single 0.33 Gy dose of irradiation for an additional 24 h. (E and F) protein levels were assayed with Western blot and expression levels as the indicated ratio (triplicate cultures). (G) Mitochondrial ROS alterations in the presence or absence of irradiation (*n* = 8 wells/group). (H-J) RAW 264.7 cells were transduced with plasmids expressing either SOD2 WT or lysine acetylation mutants targeting K68 or K122R and were cultured with RANKL for (H) 5 d or (I and J) 3 d. (H) Representative images (left) and number (right) of TRAP-positive multinucleated osteoclasts (quadruplicate cultures). (I) Osteoclast marker levels in mRNA of cultured osteoclasts measured by qPCR (triplicate cultures). (J) Mitochondrial ROS alterations (*n* = 6 wells/group). (K) Number of TRAP-positive multinucleated osteoclasts (quadruplicate cultures) derived from BMMs of 6-mo-old male *Sirt3*-KO and WT littermate mice, transduced with retroviral vectors expressing either SOD2 WT or K68R, cultured with RANKL for 3 d, and then exposed to a single 0.33 Gy dose of irradiation for an additional 2 d. (L-Q) SOD2 lysine acetylation mutants were transduced into RAW 264.7 cells as described earlier, and various respiratory parameters were evaluated: (L) basal oxygen consumption rate (OCR), (M) proton leak, (N) maximum respiration, (O) spare respiratory capacity, (P) ATP-linked OCR, and (Q) nonmitochondrial OCR after 3 d of RANKL treatment (*n* = 8-13 wells/group). Line and error bars represent mean ± SD. Statistical significance was determined using (B, F, and L-Q) *t-*test, (D, G, and K) 2-way ANOVA, or (I-J) 1-way ANOVA. All the measurements were performed in cultured BMMs pooled from 4 to 5 mice per group and repeated at least twice.

In mitochondria, the dismutation of O_2_^−^ is accelerated by mitochondrial SOD2, an enzyme traditionally associated with antioxidant protection. However, increases in SOD2 expression can increase oxidative stress under certain conditions, indicating a potential pro-oxidant role for SOD2.^32–34^ Bone marrow macrophages cultured with RANKL showed significantly higher levels of SOD2, comparable to the level of SIRT3, particularly in the late stage of osteoclastogenesis, with an identical pattern to that of SIRT3 (Figure 5A and B). SOD2 acetylation at lysine 68 increased significantly following 3 d of RANKL stimulation, which corresponded with elevated total SOD2 protein levels (Figure 5C and D). This acetylation was further enhanced in *Sirt3*-KO osteoclasts, despite a reduction in SOD2 protein levels under normal conditions. However, the extent of this hyperacetylation was modest, approximately 2-fold, corresponding to the total protein amount (Figure 5C and D). Interestingly, under IR exposure, SOD2 acetylation at lysine 68 was markedly elevated in *Sirt3*-KO osteoclasts, approximately 5-fold, compared to normal conditions, while total SOD2 protein levels exhibited a dramatic decrease (Figure 5E and F). At the same time, the levels of mitochondrial ROS increased significantly in WT osteoclasts in response to IR exposure, while the levels of mitochondrial ROS were much lower in *Sirt3*-KO osteoclasts after IR exposure (Figure 5G). If the decrease in mitochondrial ROS and bone resorption in *Sirt3*-deficient cells after IR exposure is due to hyperacetylation of SOD2, then a SOD2 acetylation-deficient mutant should restore generation of mitochondrial ROS and bone resorption to *Sirt3*-deficient osteoclasts. To test this, we created constitutively deacetylated SOD2 mutants (KR-SOD2), based on the acetylation sites (K68 and K122) identified in previous studies (Figure S3).^35^ Transient transfection was used to overexpress the mutant constructs in RAW 264.7 cells. The SOD2 K68R mutant, but not the SOD2 K122R mutant, enhanced the formation of osteoclasts (Figure 5H) and increased mRNA levels of osteoclast markers (Figure 5I). This was associated with increased ROS production (Figure 5J), as well as increases in basal respiration, proton leak, and nonmitochondrial respiration (Figure 5L-Q), similar to what was observed in irradiated osteoclasts from WT mice.^20^^,^^21^ Importantly, retroviral infection with the SOD2 K68R mutant had minimal effects on the formation of multinucleated giant osteoclasts (with more than 10 nuclei) in WT BMM cultures under IR exposure. However, constitutive deacetylation of SOD2 at K68 significantly rescued the inhibitory effects of *Sirt3* deletion (Figure 5K). A similar pattern was observed under nonirradiated conditions, although the inhibitory effects of *Sirt3* deletion were less pronounced compared to those under IR exposure (Figure S4). These findings suggest that SOD2 may modulate SIRT3-mediated mitochondrial quality control via mitochondrial ROS. Indeed, SIRT3 regulates mitochondrial quality through the direct deacetylation of SOD2 in several other tissues.^35–37^

### Pharmacological inhibition of mitochondrial ROS production replicates the effects of *Sirt3* deletion in osteoclasts

**Figure 6:**
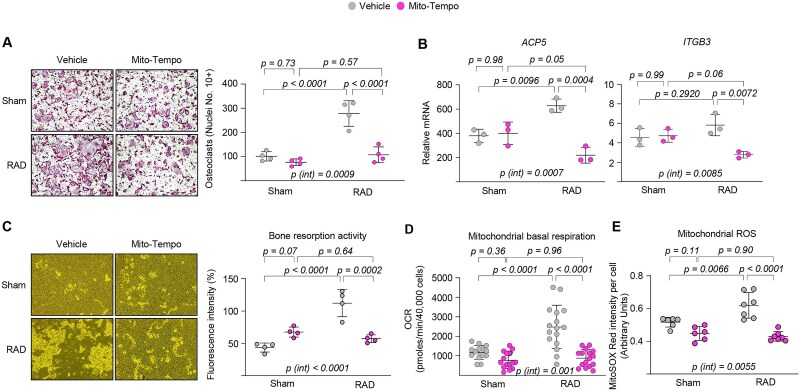
Pharmacological inhibition of mitochondrial ROS production impairs osteoclastogenesis. BMMs were harvested from 6-mo-old male C57BL/6 mice, cultured with M-CSF and RANKL for 2 d, and then subjected to a single 0.33 Gy dose of irradiation followed by (A and C) an additional 2 d or (B, D, and E) 24 h with or without Mito-TEMPO (10 μm). (A) Representative images (left) and quantification (right) of TRAP-positive multinucleated osteoclasts in quadruplicate cultures. (B) Osteoclast marker mRNA levels in cultured osteoclasts measured by qPCR (triplicate cultures). (C) Representative images (left) and quantification (right) of bone resorption activity treated with or without Mito-TEMPO (10 μm; *n* = 4 wells/group). (D) Mitochondrial basal OCR (*n* = 14-16 wells/group). (E) Mitochondrial ROS alterations in the presence or absence of irradiation (*n* = 6- to 7 wells/group). Line and error bars represent mean ± SD. Statistical significance was determined using 2-way ANOVA. All the measurements were performed in cultured BMMs pooled from 4 to 5 mice per group and repeated at least twice.

We further investigated whether mitochondrial ROS contribute to osteoclast maturation in response to IR exposure. To test how blocking mitochondrial ROS affects RANKL-induced osteoclast maturation during IR exposure, we treated BMMs with Mito-TEMPO, a mitochondrial superoxide scavenger,^38^ starting on day 3 of IR exposure and continuing until the end of the experiment on day 5. The addition of Mito-TEMPO to cultures of pre-osteoclasts decreased osteoclast formation (Figure 6A) and the expression of late/terminal osteoclast differentiation markers in the presence of IR exposure (Figure 6B). Most osteoclasts formed in the presence of Mito-TEMPO were much smaller (Figure 6A), reminiscent of the irradiated osteoclasts from *Sirt3*-KO mice. Interestingly, Mito-TEMPO alone had little effect on inhibiting mature osteoclast formation in the absence of IR exposure, suggesting that mitochondrial ROS may play a role in osteoclast maturation under certain stress conditions such as IR exposure. Notably, bone resorption was significantly reduced when Mito-TEMPO was applied during the late stages of osteoclastogenesis under IR exposure (Figure 6C). Additionally, Mito-TEMPO decreased mitochondrial respiration in osteoclasts (Figure 6D). Mito-TEMPO has minimal impact on mitochondrial ROS production under normal conditions but strongly inhibits IR-induced mitochondrial ROS production (Figure 6E). These results indicate that pharmacological inhibition of mitochondrial ROS is sufficient to mimic the effects of *Sirt3* deletion on osteoclastogenesis following IR exposure.

## Discussion

The high fracture risk associated with the loss of bone mass is one of the most common features of IR exposure in mice and humans, but the mechanisms responsible remain unclear. Although exposure to a low dose of radiation, for example, during medical treatment or during handling of radioactive material, does not cause acute radiation syndrome such as severe gastrointestinal complications and decreased numbers of hematopoietic cells, as well as widespread skin irritation, it may raise long-term risks of metabolic diseases.^39^^,^^40^ At a molecular level, the primary consequence of low- or high-dose IR exposure is mitochondrial alterations that result in cell death, carcinogenesis, or tissue degeneration.^41^^,^^42^ Herein, we provide evidence that mitochondrial SIRT3 and SOD2 contribute to the bone loss caused by low-dose IR exposure and that the adverse effects of IR exposure likely result from enhanced mitochondrial ROS production in osteoclasts. Mice exposed to 4 Gy gamma irradiation exhibit trabecular bone loss, but not cortical bone loss. The low bone mass in the trabecular bone compartment is associated with an increase in bone resorption (Figures 1-3). In the spine, osteoclast numbers do not increase and may even slightly decrease in trabecular bone following 4 Gy TBI, with CTx levels returning to baseline (Figure 3). However, our in vitro BMM cultures demonstrate that IR exposure enhances osteoclast maturation and activity, and this effect is significantly diminished by *Sirt3* deletion (Figure 4). This apparent discrepancy could be explained by the possibility that highly activated osteoclasts exposed to low-dose IR undergo apoptosis after completing their resorptive activity on the bone surface. Supporting this hypothesis, our short-term radiation exposure experiments revealed no significant change in osteoclast numbers, yet demonstrated elevated resorptive activity at this time point (Figure S2), suggesting a potential peak in osteoclast function rather than an increase in cell number. Furthermore, it is possible that we missed the precise peak time point of IR exposure–induced bone loss, which may fall between our short-term and long-term radiation exposure experiments. Given that bone resorption is a dynamic and time-sensitive process, osteoclasts that are transiently enlarged and activated may not be captured in a histological snapshot at a fixed time point. Future studies with higher temporal resolution and more frequent sampling intervals will be needed to fully delineate the kinetics of osteoclast activation and turnover following IR exposure. In contrast, mice exposed to high-dose focal radiation (ranging from 10 to 24 Gy of gamma irradiation) exhibit bone mass loss in both trabecular and cortical bone compartments, which is associated with decreases in both bone formation and bone resorption.^28^^,^^43^^,^^44^ A decrease in osteoblast number is a major contributor to cortical thinning and a decrease in trabecular bone volume after IR exposure. This, along with the finding that the deletion of *Sirt3* in mice does not alter the osteoclast or osteoblast number but decreases bone resorption in response to 4 Gy gamma irradiation exposure, strongly suggests that the IR exposure–induced cellular alterations in the skeleton vary depending on the dose of radiation and method of its delivery, and that an increase in osteoclast resorptive activity, regardless of the number of osteoclasts, contributes to the IR exposure–induced loss of trabecular bone mass. Given the sexual dimorphic role of SIRT3 in the development of osteoporosis and other diseases,^15^^,^^18^^,^^45^^,^^46^ it is possible that an increase in osteoclast activity and bone loss with IR exposure might occur predominantly in females. Furthermore, our present study was performed with mice with global loss of *Sirt3*, indicating that we cannot rule out the possibility that the skeletal effects are secondary to changes in hormones or other metabolic derangements. Therefore, further genetic studies using female mice are required to elucidate whether *Sirt3* expression and/or mitochondrial metabolism in osteoclasts promote bone resorption in the radiation-exposed skeleton.

In mitochondria, a superoxide anion radical, a major type of oxidant for ROS, is constantly produced during respiration and rapidly converted to H_2_O_2_ by SOD2, an enzyme traditionally associated with antioxidant protection. However, under stress conditions such as aging or tumor progression, an increase in SOD2 expression not only fails to provide further protection but also promotes oxidative stress in mitochondria; thus, SOD2 has an important pro-oxidant effect in cells and in vivo.^32–34^ Studies indicated that the antioxidant or pro-oxidant effect of SOD2 depends on whether there is an accumulation of FeSOD2 (iron-incorporated SOD2) when iron-to-manganese levels are high in *Sod2*-overexpressing cells. Importantly, mice accumulate FeSOD2 in the liver when fed an iron-enriched diet or a manganese-deficient diet and exhibit mitochondrial dysfunction and oxidative stress.^33^ However, the ways in which FeSOD2-mediated mitochondrial ROS accumulation contributes to skeletal homeostasis and disease have not been reported. In our prior lysine acetylome analysis of cultured osteoclasts, we observed a significant increase in the acetylation of SOD2 at lysine residue K68 in osteoclasts lacking SIRT3 during skeletal aging.^18^ Consistent with our findings, SIRT3 activates SOD2 by deacetylating lysine 68, a process closely linked to mitochondrial ROS production.^36^^,^^47^ In addition, we show here that SOD2 is a direct target of SIRT3 in osteoclasts, and SOD2 accumulates in mature osteoclasts in response to RANKL stimulation, suggesting a possible role for SIRT3 in FeSOD2 accumulation and mitochondrial ROS production. Given the significant role of SIRT3 in the development of osteoporosis under other stress conditions such as aging or estrogen deficiency,^15–18^ it is plausible that the SIRT3–SOD2 axis is not limited to IR exposure–induced bone loss. A deficiency in *Sirt3* may broadly impair osteoclast formation under conditions involving increased mitochondrial activity. To clarify this further, genetic studies using new mouse models targeting the SIRT3–SOD2 axis or other mitochondrial ROS mediators are necessary. These studies could help determine whether SIRT3-mediated deacetylation of mitochondrial proteins in osteoclasts contributes to bone resorption in radiation-exposed skeletons.

Mitophagy is a type of macroautophagy in which damaged or dysfunctional mitochondria are targeted for degradation, and genetic suppression of macroautophagy leads to the accumulation of damaged mitochondria. Studies with mice whose osteoclasts lack crucial proteins for macroautophagy revealed that macroautophagy promotes bone resorption and loss of bone mass in the context of estrogen deficiency.^48^ Our previous findings,^15^ along with the current study, show that SIRT3 may regulate mitophagy in osteoclasts. However, the mechanisms by which osteoclast mitophagy is initiated and contributes to skeletal homeostasis and disease have not been reported. Mitochondria undergoing stress, such as from mitochondrial DNA mutations, can be targeted for degradation through four primary types of mitophagy: PINK1/PARKIN-mediated mitophagy, receptor-mediated mitophagy, lipid receptor-mediated mitophagy, and piecemeal mitophagy. PINK1/PARKIN is primarily responsible for regulating ubiquitin-dependent mitophagy, whereas receptor-mediated mitophagy can be triggered in various cellular environments by different autophagy receptors that are consistently present on the outer mitochondrial membrane, such as BNIP3 and NIX.^49^ Interestingly, under stress conditions, BNIP3 and NIX may increase production of mitochondrial ROS.^50–52^ Consistent with this, osteoclast mitophagy and ROS production are suppressed in *Sirt3*-KO osteoclasts exposed to IR exposure (Figures 4 and 5). Further, SIRT3-mediated deacetylation of SOD2 may promote osteoclast differentiation and function, in part by stimulating mitophagy, which maintains the pro-osteoclastogenic levels (at least not excessive levels) of mitochondrial ROS during IR exposure–related bone loss. Indeed, we showed previously that mitochondrial ROS in osteoclasts contribute to physiological bone resorption.^14^ In contrast to the positive role of SIRT3-mediated SOD2 deacetylation in mitochondrial ROS production and osteoclast maturation, Kim et al. suggest that SIRT3 deacetylates SOD2 proteins at the same lysine residue (K68) that we identified, increasing the activity of SOD2 to reduce ROS production and inhibit osteoclast formation.^53^ However, our present study focused on a specific stress condition (IR exposure), as well as on a specific stage of osteoclastogenesis (late activation), which can be a possible explanation for the seeming discrepancy. Guo et al. reported that SOD2 is required to maintain osteoclast formation and function under static force during orthodontic tooth movement, an acute inflammatory reaction, in line with our present findings. Static force loading promotes the expression of SOD2 during osteoclastogenesis, and silencing SOD2 decreases the number of TRAP-positive multinucleated osteoclasts under the stress condition.^54^ Considering the possible switch of mitochondrial SOD2 into a pro-oxidant peroxidase under stress conditions,^32–34^ future studies with mouse models and genetic manipulation of SOD2 acetylation should elucidate the role of mitochondrial ROS and quality control in bone resorption and in mediating SIRT3 activities during IR exposure and other stress conditions.

In conclusion, our findings demonstrate that mitochondrial deacetylase SIRT3 is a key regulator of excessive bone resorption induced by IR exposure, mirroring mechanisms observed in osteoporosis pathogenesis.^15–17^ We further establish proof of principle that pharmacological inhibition of mitochondrial ROS production with Mito-TEMPO effectively mitigates the impact of IR exposure on osteoclast maturation. However, this study has certain limitations. Notably, female mice were not included, and only a single range of IR exposure was examined. Additionally, TBI—even at low doses—can induce both short- and long-term effects on the bone marrow and other organ systems, which may secondarily influence skeletal integrity. Furthermore, while our study elucidates the direct role of SIRT3 in radiation-induced bone resorption, it does not fully capture the potential indirect effects arising from osteoclast–osteoblast interactions. Future research integrating coculture experiments with primary osteoclasts and osteoblasts, as well as in vivo administration of Mito-TEMPO, could provide further mechanistic insights into the cellular cross talk involved in radiation-induced bone loss. To better isolate the skeletal effects of IR exposure, future studies should implement focal irradiation of bone, ensuring that observed bone loss is not confounded by irradiation effects on nonskeletal tissues. Despite these limitations, our study provides a valuable model for assessing the skeletal risks associated with occupational or cosmic radiation exposure. The findings presented here underscore the critical importance of mitochondrial quality control and protein acetylation in protecting against IR-induced bone deterioration. Given the well-documented clinical consequences of mitochondrial dysfunction, our findings hold promise for the development of therapeutic strategies aimed at mitigating or even preventing radiation-induced skeletal damage.

## Supplementary Material

Supplementary_Figure_1_ziaf092

Supplementary_Figure_2_ziaf092

Supplementary_Figure_3_ziaf092

Supplementary_Figure_4_ziaf092

## Data Availability

The data that support the findings of this study are available from the corresponding author upon reasonable request.
