# Supplementary figures and images for: Sirtuin-3 promotes osteoclast maturation and bone loss by regulating mitochondrial ROS production during ionizing radiation exposure
Source: JBMR Plus. 2025 May 19;9(7):ziaf092. doi: 10.1093/jbmrpl/ziaf092 (PMC12202156; doi:10.1093/jbmrpl/ziaf092)

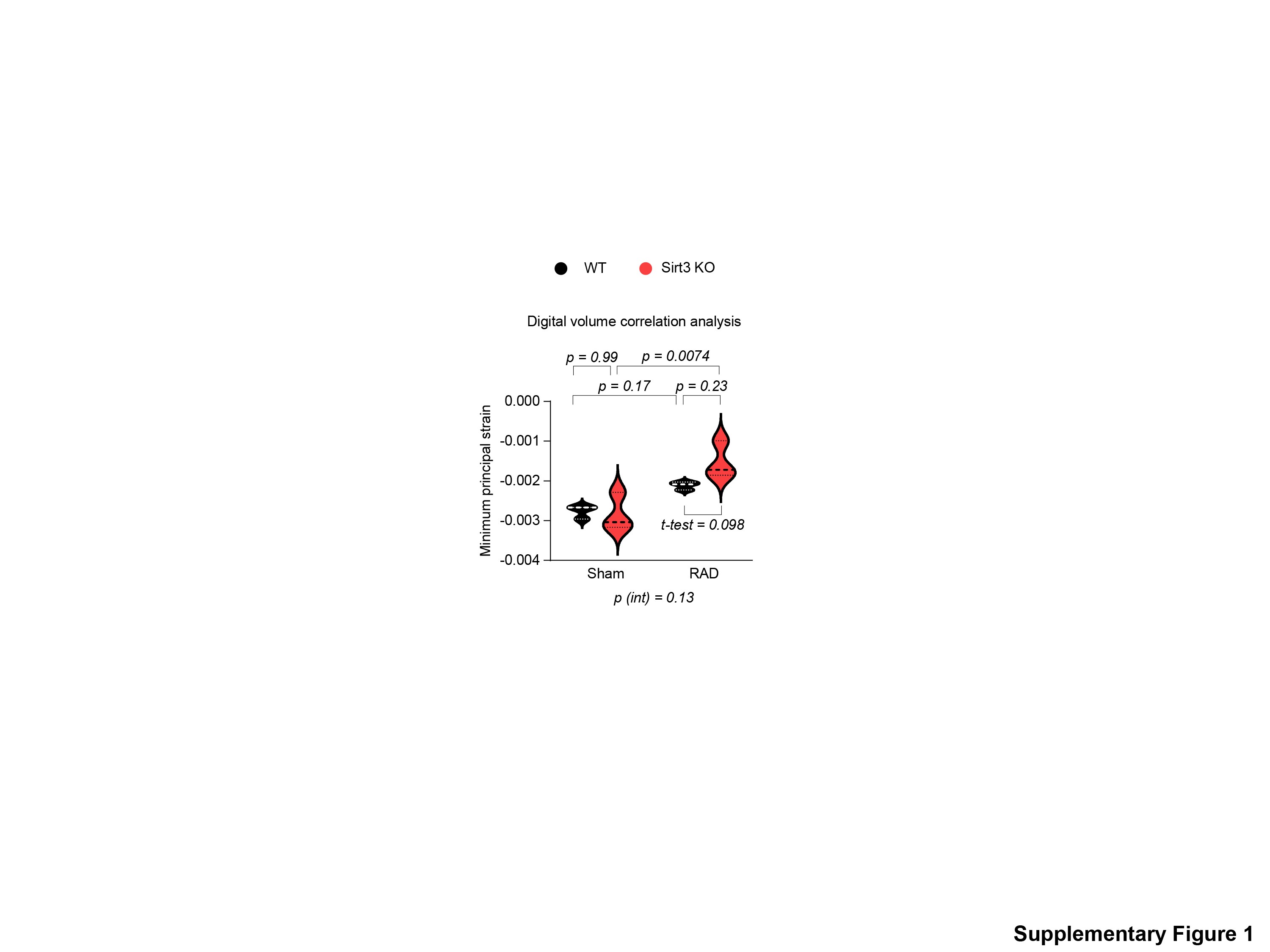

Supplement: Supplementary_Figure_1_ziaf092 [file supplementary_figure_1_ziaf092.jpeg]

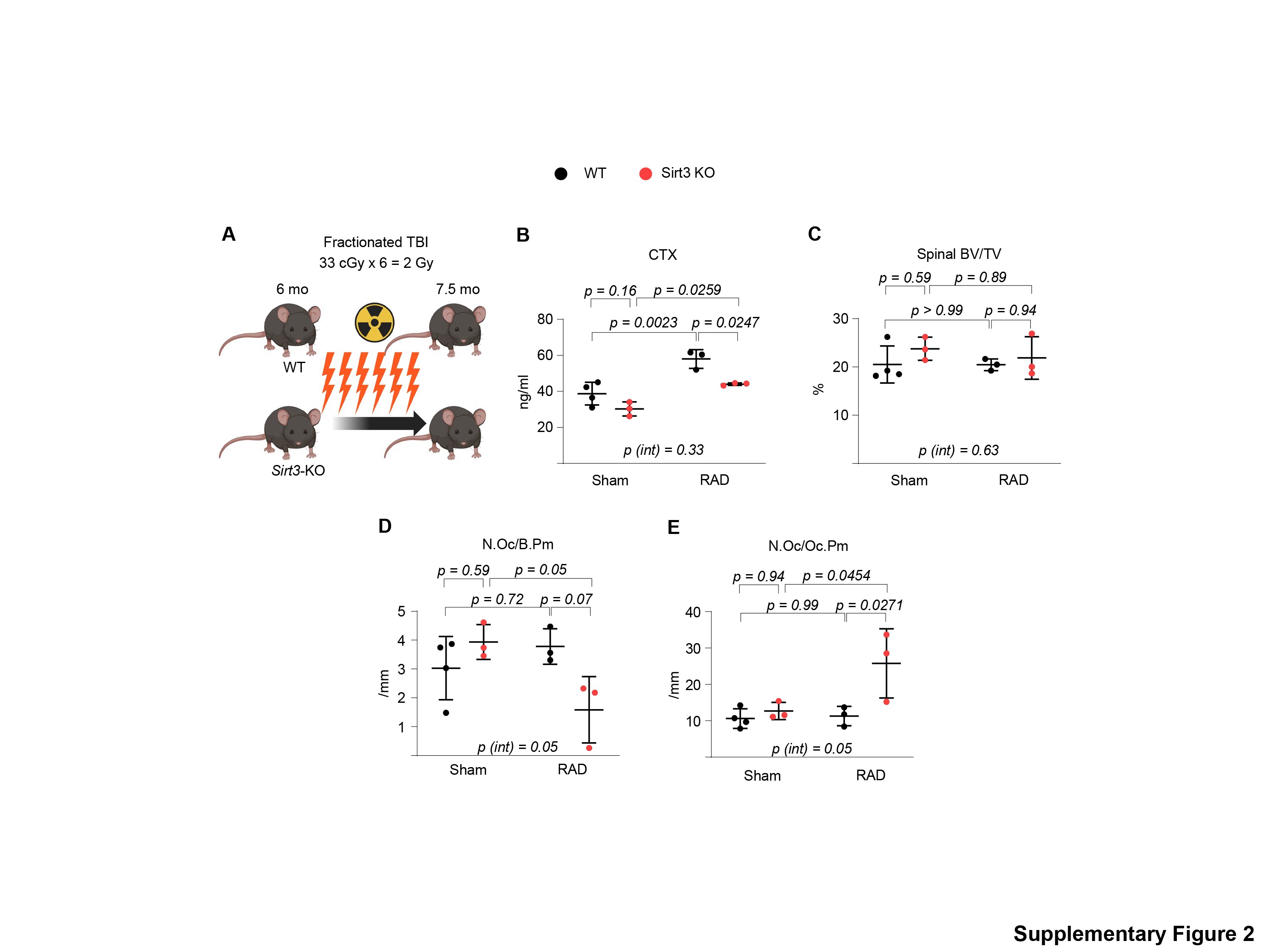

Supplement: Supplementary_Figure_2_ziaf092 [file supplementary_figure_2_ziaf092.jpeg]

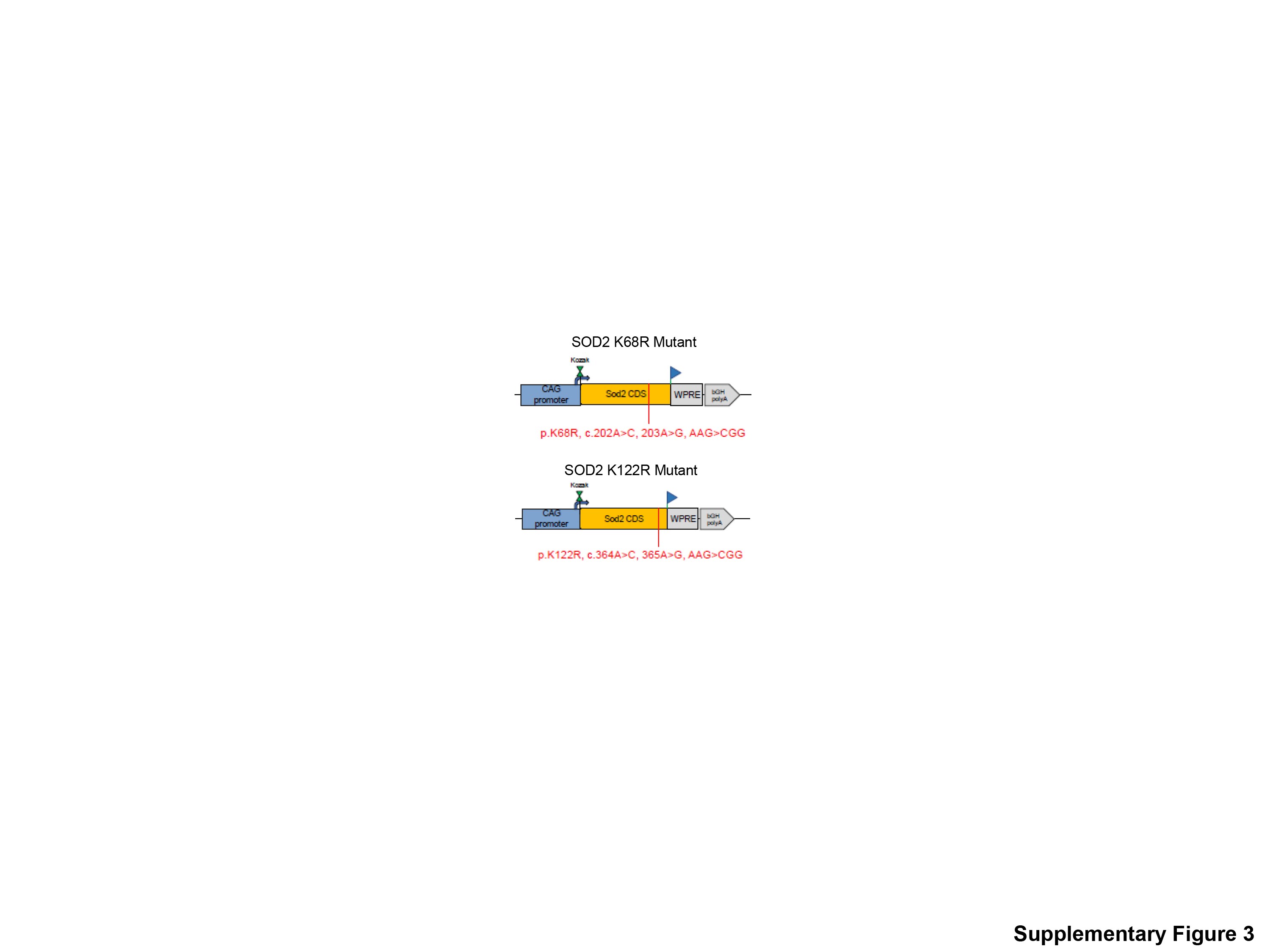

Supplement: Supplementary_Figure_3_ziaf092 [file supplementary_figure_3_ziaf092.jpeg]

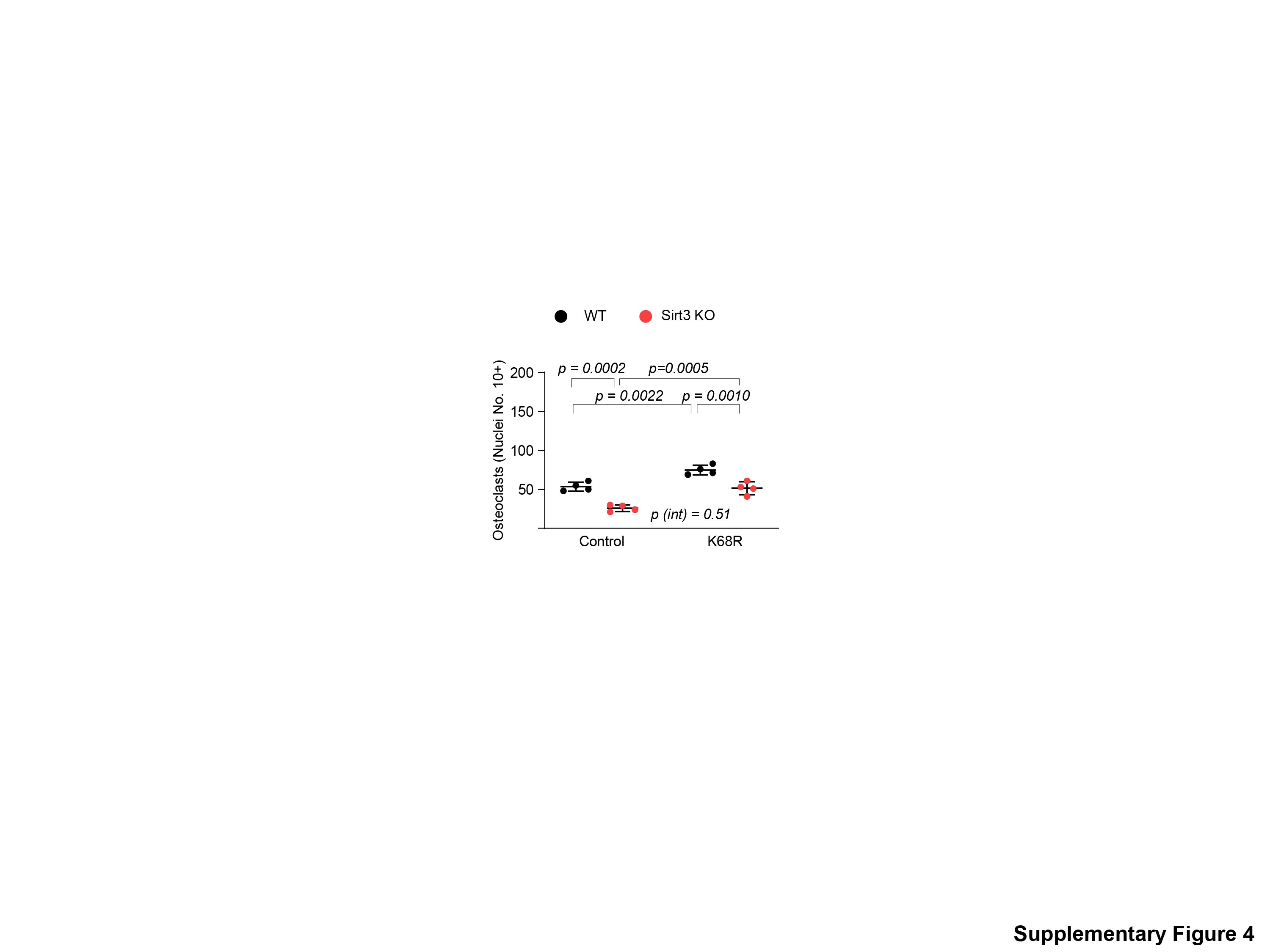

Supplement: Supplementary_Figure_4_ziaf092 [file supplementary_figure_4_ziaf092.jpeg]
